# Convergent adaptive architectures linking chemoresistance and radioresistance in chemoradiotherapy: a systems-level perspective

**DOI:** 10.3389/fonc.2026.1813711

**Published:** 2026-07-15

**Authors:** Xudong Yi, Faqiao Song, Shinsuke Nagasawa, Syogo Imai, Hiroyuki Arimatsu, Teppei Yamaoka, Kenjiro Shibata, Ryuya Hayashida, Aoi Okuno, Tomo Urano, Haruto Takahashi, Zhaohua Chang, Anji Wang, Kaizhen Yang, Hengmao Zhang, Haobo Zhao, Junko Takahashi

**Affiliations:** 1 Graduate School of Information, Production and Systems, Waseda University, Kitakyushu, Japan; 2 Department of Radiology, Graduate School of Medical Science, Kyoto Prefectural University of Medicine, Kyoto, Japan

**Keywords:** 5-fluorouracil, chemoradiotherapy, cisplatin, cross-resistance, mitochondrial metabolism, radioresistance, redox adaptation, temozolomide

## Abstract

**Background:**

Chemoradiotherapy (CRT) remains a cornerstone of treatment for multiple solid malignancies; however, durable disease control is frequently limited by the emergence of resistance. While chemoresistance and radioresistance have traditionally been investigated as modality-specific phenomena, accumulating evidence suggests substantial biological convergence between them.

**Methods:**

This review synthesizes mechanistic insights from drug-specific resistance models and proposes a convergence framework in which cross-resistance arises from partially shared adaptive resistance mechanisms rather than simple overlap in initial DNA lesions.

**Result:**

Across major CRT backbone agents—including platinum compounds, temozolomide, and 5-fluorouracil—resistance commonly arises through multilayered adaptive programs rather than isolated lesion-specific alterations. Reinforcement of DNA damage response (DDR) capacity, replication stress management, mitochondrial metabolic remodeling, antioxidant buffering, and stabilization of stem-like cell states collectively reduce the probability that genotoxic insults are converted into lethal damage. Although the primary DNA lesions induced by chemotherapy and radiotherapy differ, resistant tumors often appear to converge on shared adaptive infrastructures that modulate damage sensing, processing, and fate determination. Mitochondrial functional plasticity and redox regulation may contribute to these shared resistance-associated processes by influencing reactive oxygen species (ROS) buffering and cellular stress tolerance across treatment modalities.

**Conclusion:**

Targeting shared adaptive resistance mechanisms—rather than single damage pathways—may represent a strategic direction for overcoming resistance in CRT-treated cancers.

## 1 Introduction

Chemoradiotherapy (CRT) has become a cornerstone of curative-intent treatment for many locally advanced malignancies, integrating cytotoxic chemotherapy with radiotherapy to improve local control and survival outcomes. However, despite advances in delivery techniques and combined modality strategies, a significant proportion of patients who initially achieve clinical responses ultimately experience treatment failure, largely due to the emergence of chemoresistance and/or radioresistance. Clinical observations that tumors resistant to cytotoxic agents often exhibit reduced sensitivity to ionizing radiation suggest that these resistance phenotypes may not be entirely independent and could arise from overlapping biological processes.

**Figure 1:**
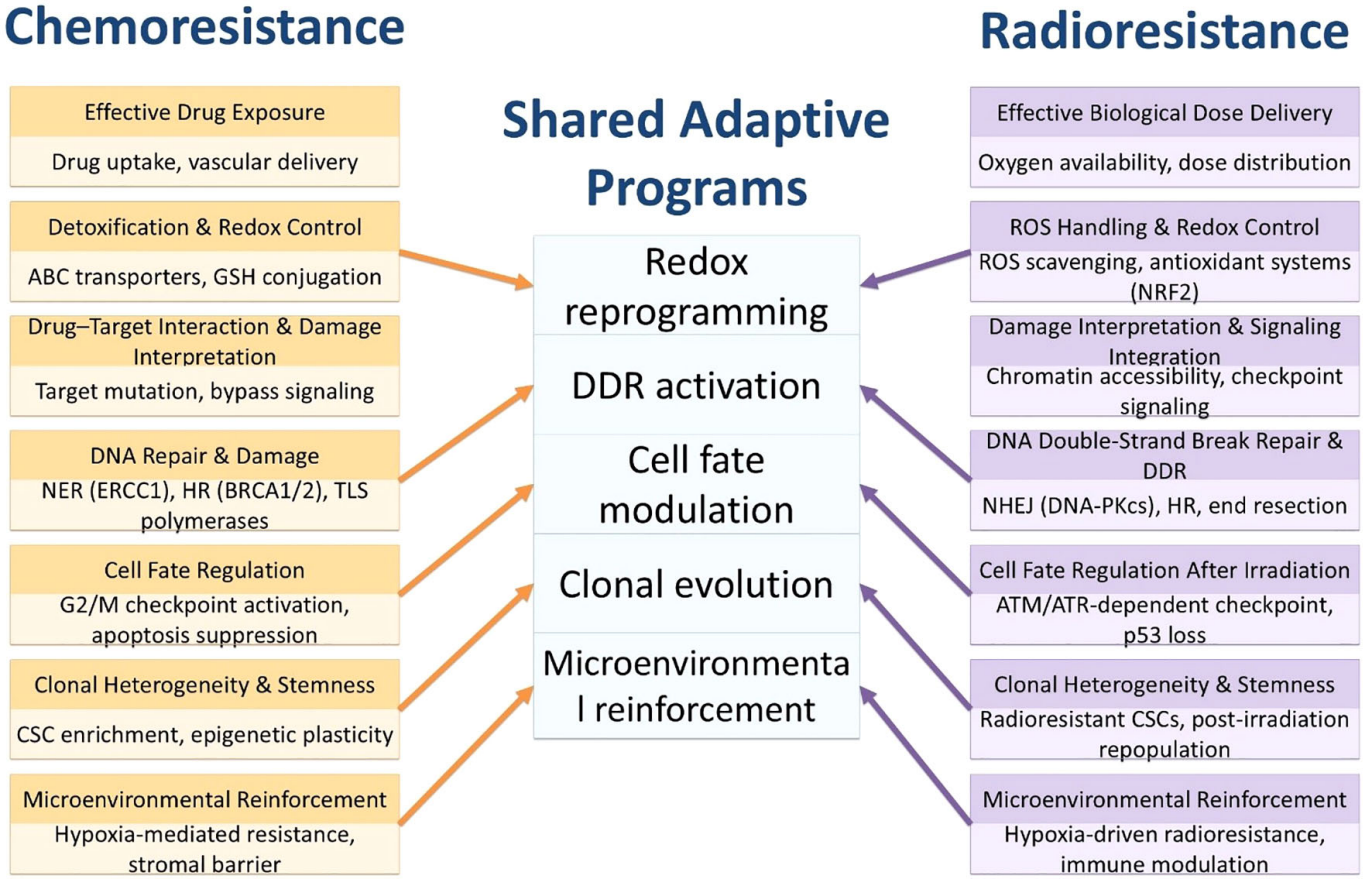
Conceptual model illustrating the partial dissociation and shared determinants of chemoresistance and radioresistance. The left and right panels summarize representative biological layers associated with chemoresistance and radioresistance, respectively. Chemoresistance-related modules include altered drug exposure, detoxification/redox buffering, drug–target interaction, DNA repair capacity, cell fate regulation, clonal heterogeneity/stemness, and microenvironmental reinforcement. Radioresistance-related modules include effective biological dose delivery, ROS handling, damage signaling integration, DNA double-strand break repair and DDR activation, irradiation-associated cell fate regulation, stemness-associated repopulation, and microenvironmental adaptation. Despite distinct initiating mechanisms, both resistance phenotypes may partially converge on shared adaptive programs involving redox reprogramming, DDR activation, cell fate modulation, clonal evolution, and microenvironmental reinforcement. Colored arrows indicate conceptual convergence from modality-specific resistance layers toward overlapping adaptive resistance mechanisms rather than strict one-to-one mechanistic correspondence. ABC transporter, ATP-binding cassette transporter; ATM, Ataxia telangiectasia mutated; ATR, Ataxia telangiectasia and Rad3-related protein; BRCA1/2, Breast cancer type 1/2 susceptibility proteins; CSC, Cancer stem cell; DDR, DNA damage response; DNA-PKcs, DNA-dependent protein kinase catalytic subunit; ERCC1, Excision repair cross-complementation group 1; GSH, Glutathione; HR, Homologous recombination; MHEJ, Non-homologous end joining; NER, Nucleotide excision repair; NRF2, Nuclear factor erythroid 2-related factor 2; p53, Tumor protein p53; ROS, Reactive oxygen species; TLS, Translesion synthesis.

Traditionally, chemoresistance and radioresistance have been regarded as distinct entities — the former largely attributed to drug-specific mechanisms such as efflux pumps, DNA repair alterations, and target modifications, whereas the latter has been associated with factors such as radiation dose distribution, tumor hypoxia, and DNA damage response (DDR) capacity. However, accumulating evidence from studies of radioresistance indicates that molecular and cellular determinants including enhanced DNA damage repair, dysregulated cell cycle control, hypoxia, and stem-like tumor cell subpopulations may also contribute to resistance across multiple treatment modalities (1, 2). Collectively, these observations raise the possibility that common downstream mechanisms may underlie both chemoresistance and radioresistance, as conceptually illustrated in **Figure 1**.

Despite these insights, the current literature remains fragmented, with many mechanistic studies focusing on isolated pathways or specific treatment contexts. As a result, a comprehensive conceptual framework that systematically integrates resistance mechanisms across chemotherapy and radiotherapy remains lacking. There is a need to understand under what conditions cross-resistance arises and how shared biological pathways can inform treatment design and optimization.

To address these gaps, this review reconceptualizes chemoresistance as a set of clinically observable “resistance modules” rather than isolated drug-specific mechanisms. These modules include inadequate drug exposure, detoxification and drug efflux, DNA repair and damage tolerance, cell fate alterations, intratumoral heterogeneity and stemness, and the tumor microenvironment influences. Within this modular framework, we then examine radioresistance through the same conceptual lens, revealing partial overlap in downstream determinants such as DDR reinforcement, hypoxia-associated adaptation, and stem-like plasticity, thereby supporting the existence of shared adaptive resistance infrastructures that transcend individual treatment modalities.

Emerging studies also implicate metabolic and oxidative stress adaptations in shared resistance-associated responses, while mitochondrial functional states may influence cellular adaptation to both chemotherapy and radiotherapy, including redox homeostasis and energy metabolism (3, 4). By synthesizing these findings, we aim to provide a unified understanding of CRT resistance and lay the groundwork for the rational redesign of CRT strategies that account for potential cross-resistance.

## 2 Literature search and study selection

This review was conducted as a narrative synthesis of the literature focusing on treatment resistance in CRT. Relevant publications were identified primarily through searches of the PubMed database. The search strategy combined drug-related terms [e.g., cisplatin, temozolomide (TMZ), 5-fluorouracil (5-FU)], radiotherapy-related terms (e.g., radiotherapy, irradiation, radiation response), resistance-related keywords, and cancer types. Particular attention was given to platinum-based CRT while also including representative agents commonly used in combination with radiotherapy. Titles and abstracts retrieved from the initial search were screened to exclude studies clearly unrelated to CRT or treatment resistance. Full texts of potentially relevant articles were subsequently evaluated for conceptual relevance and mechanistic contribution to the present review.

Although the literature search followed a structured strategy, this article does not constitute a formal systematic review and did not adhere to PRISMA-based reporting criteria. Final study selection was guided by mechanistic relevance, clinical applicability to CRT, and contribution to understanding chemoresistance, radioresistance, or cross-resistance mechanisms. Both preclinical (*in vitro* and *in vivo*) and clinical studies were considered. Original research articles were prioritized for mechanistic and clinical interpretation, while review articles were used primarily to provide contextual background. Studies focusing exclusively on radiotherapy without systemic treatment relevance or lacking direct association with treatment resistance mechanisms were excluded. Selected studies were categorized according to drug class, cancer type, resistance phenotype (chemoresistance and/or radioresistance), and level of evidence (preclinical or clinical). Clinical implications were noted only when explicitly discussed in the original publication. This categorization was intended to support conceptual integration rather than quantitative meta-analysis. As a narrative review, some degree of selection bias may remain unavoidable, particularly regarding emphasis on representative mechanistic studies and convergent resistance models.

## 3 Clinical burden of resistance in CRT

CRT has led to substantial improvements in survival and local control across multiple malignancies. Nevertheless, treatment failure remains frequent despite initially favorable responses, and resistance remains a major determinant of long-term outcomes across multiple cancer types (**Table 1**).

**Table 1:** Clinical burden of CRT resistance.

Drug backbone	Cancer type	Treatment setting	Key resistance issue	Representative clinical outcome	Current clinical treatment strategies	Ref.
Cisplatin	Cervical cancer (LACC)	CCRT	Limited cisplatin benefit; early relapse after CCRT	5-year OS: 70%; clinically evident early-relapse subgroup	Cisplatin-based CCRT ± induction chemotherapy	(19–21)
Cisplatin	Nasopharyngeal cancer	CCRT	Persistent/recurrent disease after cisplatin-based CRT	5-year OS: 70% in phase III trial; substantial recurrence rate	Cisplatin-based CCRT ± induction chemotherapy	(22–24)
Cisplatin and/or Carboplatin based	Stage III NSCLC	CCRT → durvalumab	Poor disease control after platinum-based CRT	5-year OS: 43% (durvalumab arm); >50% progression/death within 5 years	Platinum-based CRT → durvalumab consolidation	(25–27)
Carboplatin-based CRT	Esophageal cancer	Neoadjuvant CRT	Frequent primary resistance to carboplatin-based CRT	Pathologic complete response: 29% overall (≈49% SCC, ≈23% adenocarcinoma)	Carboplatin/paclitaxel CRT → surgery; definitive CRT	(6, 28)
TMZ	Glioblastoma	RT + TMZ	MGMT-linked resistance; near-universal recurrence	Median OS: 14–15 months; most patients recur	Maximal resection + RT + concurrent/adjuvant TMZ	(10, 29)
5-FU	Gastrointestinal cancers (rectal, esophageal subsets)	CCRT or neoadjuvant CRT	Variable response to 5-FU based CRT	Variable pathologic response; no uniform survival benefit across disease stages	5-FU-based CRT ± surgery (disease-stage dependent)	(30, 31)

5-FU, 5-fluorouracil; CCRT, concurrent chemoradiotherapy; CRT, chemoradiotherapy; LACC, locally advanced cervical cancer; NSCLC, non-small cell lung cancer; OS, overall survival; RT, radiotherapy; SCC, squamous cell carcinoma; TMZ, temozolomide; MGMT, O^6^-methylguanine-DNA methyltransferase.

### 3.1 Platinum-based CRT (cisplatin and carboplatin)

Platinum-based CRT represents the backbone of concurrent treatment in several solid tumors, including stage III non-small cell lung cancer (NSCLC), locally advanced cervical cancer, esophageal cancer, and nasopharyngeal carcinoma. Despite significant improvements in survival with concurrent platinum-based CRT and subsequent consolidation therapies, a substantial proportion of patients develop progression or relapse (5, 6). In NSCLC, for example, more than half of patients experience progression or death within five years of definitive concurrent CRT (7). Similarly, in cervical and nasopharyngeal cancers, cisplatin-based CRT achieves high initial response rates but fails in a clinically distinct subset characterized by non-response or early recurrence (8, 9). These observations indicate that platinum resistance not only compromises systemic control but may also attenuate the therapeutic synergy between chemotherapy and radiotherapy.

### 3.2 TMZ-based CRT

TMZ-based CRT, the standard of care for glioblastoma, provides another paradigm. Although radiotherapy combined with TMZ prolongs survival compared with radiotherapy alone, median overall survival remains approximately 14–15 months, and recurrence is nearly universal (10, 11). A considerable fraction of tumors demonstrates primary chemoresistance or rapid acquisition of resistance, frequently associated with O^6^-methylguanine-DNA methyltransferase (MGMT)-mediated repair of O^6^-methylguanine lesions (12). In this setting, chemoresistance and radioresistance appear closely intertwined within an intrinsically aggressive biological context.

### 3.3 5-FU-based CRT

5-FU-containing CRT regimens, widely used in gastrointestinal malignancies, similarly illustrate the complexity of resistance. In esophageal cancer, for example, neoadjuvant CRT achieves pathologic complete response in only a subset of patients, while others exhibit primary resistance to the combined modality (13, 14). The degree to which chemoresistance translates into reduced radiosensitivity varies across tumor types and treatment contexts, underscoring the heterogeneity of resistance phenotypes (15). Clinically, resistance manifests as primary non-response, early relapse during therapy, or recurrence after an initial response. From a radiobiological perspective, resistance may arise through partially overlapping but not fully equivalent mechanisms. Loss of chemotherapy-mediated radiosensitization may reduce the additive or synergistic effects of CRT, while intrinsic radioresistance reduces the efficacy of radiotherapy itself (16, 17). In clinical practice, these processes frequently coexist, and their relative contributions likely vary across tumor types and treatment contexts (18).

Taken together, these observations suggest that resistance in CRT is not merely an incidental consequence of treatment failure but reflects interconnected and potentially convergent biological adaptations that influence both chemoresistance and radioresistance. This conceptual overlap highlights the need for an integrated mechanistic framework to explain how chemoresistance and radioresistance converge within the same tumor ecosystem.

## 4 Mechanistic classification of chemoresistance

**Figure 2:**
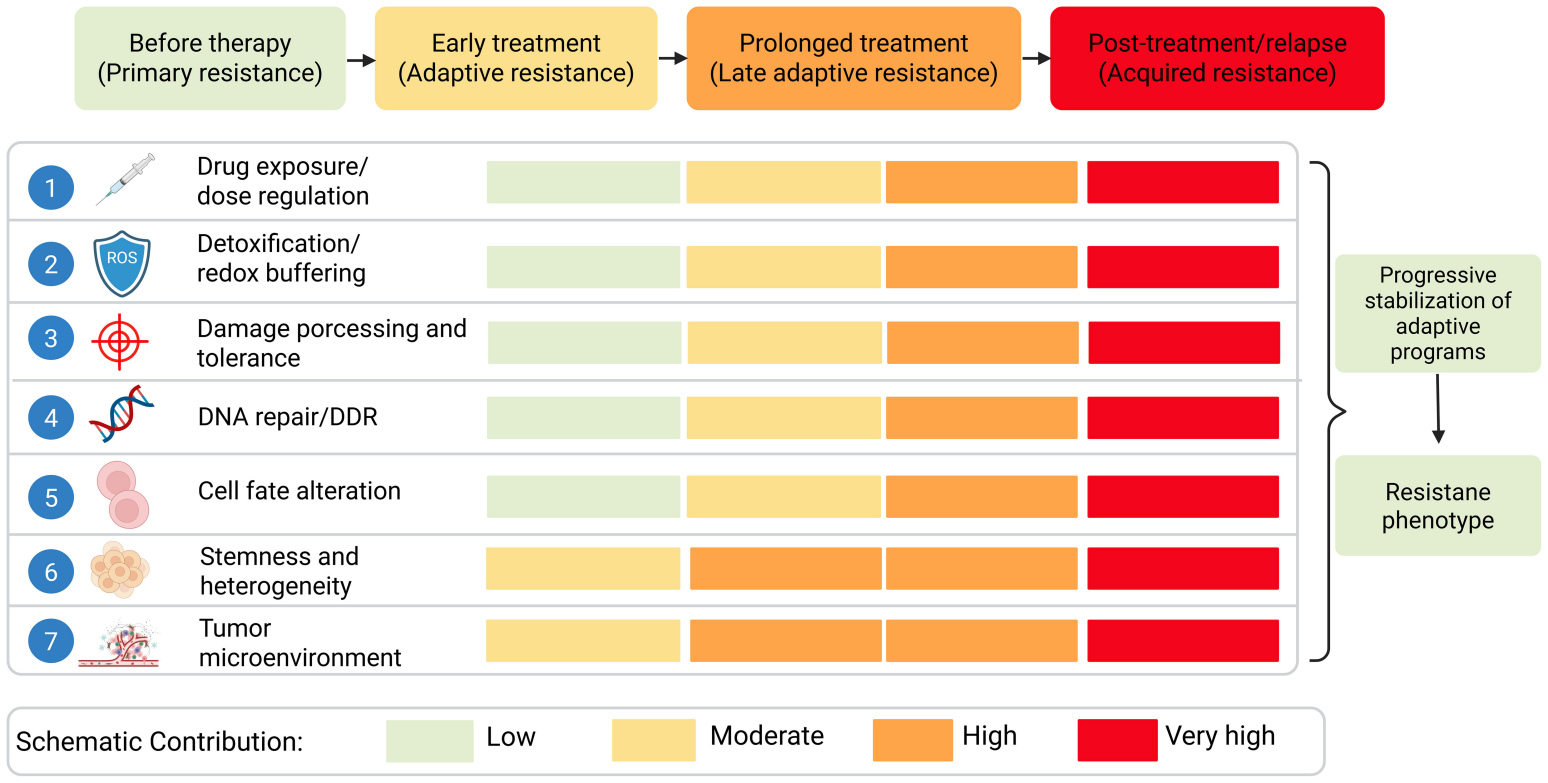
Temporal evolution and stabilization of adaptive chemoresistance during therapeutic exposure. This schematic illustrates the progressive development of chemoresistance during cancer therapy, spanning primary resistance before treatment initiation, adaptive resistance during early and prolonged treatment exposure, and acquired resistance following relapse. The color-coded bars indicate the relative contribution of each resistance-associated module over time, ranging from low to very high contribution levels. The illustrated modules include effective drug exposure/dose regulation, detoxification and redox buffering, damage processing and tolerance, DNA repair and DDR activation, cell fate alteration, stemness/clonal heterogeneity, and tumor microenvironmental reinforcement. Early adaptive states are characterized by heterogeneous and partially reversible responses, whereas prolonged treatment exposure progressively reinforces multiple resistance-associated programs, ultimately contributing to establishment of a persistent chemoresistant phenotype. The schematic contribution levels are intended to represent conceptual trends in resistance evolution rather than quantitative measurements or strict temporal kinetics.

Chemoresistance should not be interpreted as a single molecular abnormality but rather as a temporally structured biological process. For mechanistic clarity, resistance can be conceptualized along a temporal axis comprising the following stages: (i) primary resistance, which pre-exists prior to therapeutic exposure; (ii) adaptive resistance, a reversible state induced during treatment; and (iii) acquired resistances, a stabilized phenotype emerging through clonal selection under sustained therapeutic pressure, as illustrated in **Figure 2**.

To provide a clinically applicable framework, chemoresistance can be organized into several conceptually interconnected resistance modules that extend across individual drug classes. This classification provides a common conceptual structure for subsequent drug-specific discussion and for comparison with radioresistance in the context of CRT.

### 4.1 Insufficient effective exposure

Therapeutic failure may occur independently of intrinsic cellular drug sensitivity when effective concentrations of anticancer agents fail to reach tumor cells. Abnormal tumor vasculature, elevated interstitial pressure, and blood–brain barrier restrictions can limit intratumoral drug delivery. Even after cellular uptake, intracellular sequestration or altered subcellular distribution may prevent adequate target engagement (32). This module represents the first functional layer of resistance and is often difficult to predict based solely on molecular profiling.

### 4.2 Detoxification and efflux

Following successful intracellular delivery, effective cytotoxicity may still be attenuated by detoxification and active efflux mechanisms. Glutathione-mediated conjugation and enzymatic detoxification represent major mechanisms of metabolic resistance, particularly for platinum agents and other DNA-damaging agents. In addition, ATP-dependent efflux transporters, exemplified by P-glycoprotein (ABCB1), reduce intracellular drug accumulation, thereby lowering effective cytotoxic exposure (33–35). These detoxification processes are closely linked to redox homeostasis. Glutathione metabolism and NAD(P)H-dependent reduction systems are integrated with cellular energy metabolism, and redox adaptation has been shown to influence treatment response in multiple settings (36).

### 4.3 Altered drug –target interaction

Even when intracellular drug exposure is sufficient, resistance may still arise if drug-target interactions are altered. Altered expression levels of drug targets, target mutations, alternative functional isoforms, or activation of bypass signaling pathways can attenuate drug efficacy. In DNA-targeting therapies, the concept of “damage tolerance” explains how cells may continue proliferating despite persistent DNA lesions (37–39).

### 4.4 DNA repair and damage tolerance

DDR pathways constitute a central and recurrent resistance mechanism for DNA-damaging agents. Repair mechanisms remove DNA lesions, whereas damage tolerance mechanisms permit cell survival without fully resolving damage. The clinical relevance of MGMT-mediated repair in glioblastoma was demonstrated by Hegi et al. (40), who showed that MGMT promoter methylation predicts benefit from TMZ therapy. DNA repair is energetically demanding and dependent on ATP and NAD^+^ availability. Thus, cellular metabolic state can influence repair capacity. The link between homologous recombination deficiency and treatment sensitivity was illustrated in BRCA2-deficient cells, where impaired repair increased susceptibility to DNA-damaging agents (41). Because radiation also induces DNA double-strand breaks (DSBs), DDR represents a major mechanistic intersection between chemoresistance and radioresistance.

### 4.5 Cell fate alteration

Beyond damage induction and repair capacity, therapeutic outcome is also shaped by how tumor cells interpret and execute stress responses. Tumor cells may evade apoptosis, undergo senescence, or survive mitotic catastrophe. The clinical significance of apoptosis resistance was underscored by early studies demonstrating that defects in cell death pathways contribute directly to therapeutic resistance (42). Such alterations may contribute to the clinical dissociation between initial tumor shrinkage and long-term disease control.

### 4.6 Intratumoral heterogeneity and stemness

Tumors contain subpopulations with intrinsic treatment resistance (43). Cancer stem-like cells (CSC) have been shown to preferentially activate DNA damage checkpoints and survive radiation exposure (44). Selection of these subpopulations under therapeutic pressure can lead to the enrichment and stabilization of resistant clones at the population level. This module may contribute to both primary and acquired resistance across treatment modalities.

### 4.7 Tumor microenvironment

Chemoresistance is not solely determined by intrinsic properties of tumor cells but is also shaped by the tumor microenvironment. Hypoxia not only limits drug distribution but also modulates DDR and cell fate programs, including the maintenance of stem-like states. In addition, inflammatory and immune conditions constitute critical modulators of therapeutic response (45–48).

### 4.8 Summary

Chemoresistance emerges from multiple interacting resistance mechanisms operating across different biological levels, ranging from insufficient drug exposure to microenvironmental influences. This framework provides a basis for understanding treatment failure and for analyzing potentially shared mechanisms with radioresistance in CRT. Although presented separately for conceptual clarity, these resistance modules are highly interconnected and frequently co-evolve during therapeutic adaptation, collectively shaping therapy-tolerant tumor states.

## 5 Mechanistic classification of radioresistance

Radiotherapy is a local treatment modality whose mode of exposure and mechanism of action differ fundamentally from systemic chemotherapy. Nevertheless, in clinical practice, local tumor control may fail despite the delivery of an apparently sufficient physical radiation dose. Such cases reflect reproducible biological mechanisms of radioresistance. Radioresistance, like chemoresistance, should not be regarded as a single molecular abnormality but rather as a temporally structured and biologically hierarchical process (16, 49).

In this section, radioresistance is organized using conceptual modules corresponding to those applied to chemoresistance. This parallel framework aims to clarify both shared resistance infrastructures and modality-specific determinants, thereby providing a basis for understanding mechanistic intersections and potential cross-resistance in combined CRT.

### 5.1 Insufficient effective biological dose

The first layer of radioresistance arises when lethal biological effects are not uniformly delivered to all tumor cells. Even when the prescribed physical dose is administered, heterogeneous dose distribution, constraints imposed by normal tissue tolerance, tumor geometry, and deep anatomical location may prevent the uniform induction of lethal damage throughout the tumor mass (50, 51). In addition, intratumoral hypoxia reduces the oxygen enhancement effect, thereby attenuating the fixation of radiation-induced DNA damage despite identical physical doses (52). This module corresponds conceptually to “insufficient effective exposure” in chemoresistance and represents the initial clinical and biophysical layer of radioresistance.

### 5.2 ROS handling and redox buffering

A substantial fraction of the indirect biological effects of ionizing radiation is mediated by reactive oxygen species (ROS), which contribute to DNA damage formation (53). Although antioxidant enzyme induction and glutathione metabolism have long been regarded as central to ROS handling, experimental manipulation of intracellular glutathione levels demonstrates only limited effects on radiation survival. These findings suggest that glutathione alone does not fully account for endogenous radioprotection and support a more complex redox buffering architecture involving protein thiols, thioredoxin systems, and other reductive pathways (54). Consistent with this broader view, studies using clinically relevant radioresistant (CRR) cells have shown that radioresistant phenotypes are characterized by decreased mitochondrial membrane potential (ΔΨm) and reduced oxygen consumption, and that restoration of mitochondrial function accompanies recovery of radiosensitivity (55). These findings indicate that radioresistance may be partially reversible and metabolically regulated.

Collectively, these observations suggest that radioresistance cannot be explained solely by differences in DNA damage induction or antioxidant scavenging capacity. Instead, broader metabolic state and redox regulation may influence how radiation-induced damage becomes biologically fixed.

### 5.3 Altered damage effectiveness and damage tolerance

Unlike most targeted chemotherapies, radiotherapy does not rely on a single defined molecular target; rather, DNA DSBs are widely regarded as the principal lethal lesions (56). However, quantitative DSB measurements and repair kinetics do not always strictly predict clonogenic survival (57, 58). This module addresses how radiation-induced DNA lesions are interpreted and translated into biological consequences at the cellular level. Resistance in this context can be defined as a state in which comparable levels of induced damage fail to culminate in irreversible cell death, reflecting altered damage processing, signaling integration, or execution of cell death programs. This phenomenon is exemplified in CRR models, in which persistent DNA damage markers such as γH2AX coexist with sustained clonogenic survival (58). Conceptually, this corresponds to “altered drug–target interaction and damage tolerance” in chemoresistance and is closely linked to downstream DDR signaling and checkpoint adaptation, cell cycle checkpoint control, and cell fate determination (59).

Importantly, this framework extends beyond DNA repair capacity alone and includes mechanisms that modulate how damage signals are interpreted and integrated into survival or death decisions.

### 5.4 DNA DSB repair and DDR

A major mechanistic axis of radioresistance has traditionally been attributed to enhanced DSB repair capacity, particularly through non-homologous end joining (NHEJ)-associated factors such as DNA-PK, and activation of DDR signaling pathways (60). Following irradiation, NHEJ and HR are rapidly mobilized to repair or neutralize lethal DNA lesions. Increased efficiency, accuracy, or persistence of these pathways has long been considered a principal determinant of radioresistance (61). However, accumulating evidence indicates that DDR signaling may be influenced by factors beyond repair enzyme abundance alone. For example, Sirt3 has been implicated in modulation of ATM–Chk2 signaling pathways (62).

DNA repair processes are ATP- and NAD^+^-dependent and require coordinated chromatin remodeling and signaling cascades (63, 64). Therefore, cellular energy metabolism may influence both the efficiency and fidelity of DNA damage repair. Accordingly, radioresistance may involve metabolic regulation of DDR pathways rather than simple upregulation of repair enzymes.

### 5.5 Cell fate alteration after irradiation

DNA damage does not inevitably culminate in apoptotic cell death. Following irradiation, tumor cells may undergo apoptosis, mitotic catastrophe, senescence, transient growth arrest, or persist in aberrant multinucleated or polyploid states (65–68). These diverse outcomes reflect the post–DNA damage response (post-DDR) execution layer of radioresponse, in which damaged cells either commit to irreversible elimination or transition into survival-associated states.

Experimental studies demonstrate that ionizing radiation can induce accelerated senescence in p53-proficient tumor cells rather than immediate apoptotic death (65, 66). In parallel, irradiation frequently triggers a transient G2 arrest that may subsequently be resolved, allowing a subset of cells to re-enter the cell cycle (67). Moreover, irradiated tumor cells can survive in highly abnormal multinucleated or polyploid states for prolonged periods, retaining metabolic activity and, in some cases, clonogenic competence (68). Taken together, these findings indicate that radioresistance at the cellular level may arise when irradiated cells preferentially enter non-lethal fates such as senescence or reversible arrest, thereby avoiding apoptotic or mitotic elimination (68).

Clinically, transient tumor regression after radiotherapy may mask the persistence of a subpopulation of damaged yet viable cells that retain clonogenic potential and ultimately drive tumor repopulation. This pattern is consistent with alterations in p53-dependent death commitment and treatment responsiveness (42), and may also involve checkpoint recovery mechanisms and activation of pro-survival signaling pathways that shift the balance away from irreversible elimination and toward survival adaptation.

Thus, radioresistance at this level is not determined solely by the magnitude of DNA damage or repair efficiency, but by cellular decision thresholds that govern commitment to cell death versus survival adaptation within the post-DDR execution framework.

### 5.6 Intratumoral heterogeneity and stemness

Tumors are not homogeneous cellular populations but consist of subclonal compartments that differ in proliferative capacity, metabolic state, and DNA repair proficiency (69). Within this heterogeneous architecture, specific subpopulations exhibit intrinsic or adaptive radioresistance (44). CSC and slow-cycling tumor cells frequently display enhanced DDR activity, altered redox regulation, and increased capacity for survival following irradiation (44, 70, 71). These properties enable them to repopulate the tumor mass after treatment.

This module represents the population-level layer of radioresistance, in which cellular heterogeneity determines the probability that resistant subclones survive and expand (72, 73). Radiation-tolerant persister populations further contribute to recurrence dynamics (74). Importantly, metabolic and mitochondrial features may be preferentially enriched within these resistant subpopulations (75).

### 5.7 Tumor microenvironment

The tumor microenvironment constitutes a higher-order regulatory layer that modulates the preceding modules of radioresistance. Among microenvironmental determinants, hypoxia remains the most extensively validated factor associated with reduced radiosensitivity. This phenomenon reflects attenuation of the oxygen enhancement effect, originally described by Gray and colleagues (76), and has been corroborated in clinical settings, where hypoxia imaging prior to radiotherapy predicts inferior treatment outcomes (77, 78).

Beyond oxygen tension, stromal signaling, inflammatory mediators, immune contexture, and vascular architecture collectively influence radiation-induced cell death, DNA repair signaling, and tumor repopulation (79–83). In particular, radiation-induced antitumor efficacy has been shown to depend on type I interferon signaling and T cell–mediated immune responses, underscoring the contribution of host immunity to treatment outcome. By modulating oxygenation, perfusion, cytokine dynamics, and immune activation states, these extrinsic determinants can alter the effective biological dose delivered to tumor cells (Module 5.1) and reshape downstream cellular responses, including DNA damage repair, stress adaptation, and repopulation processes (Modules 5.2–5.5).

In this framework, radioresistance emerges not solely from tumor-intrinsic molecular alterations but also from dynamic interactions between tumor cells and their surrounding microenvironment. The microenvironment may therefore influence the development and maintenance of radioresistant phenotypes *in vivo*.

### 5.8 Summary

In this section, radioresistance has been organized into seven interconnected modules spanning physical, cellular, population, and microenvironmental layers. These include insufficient effective biological dose, redox regulation, DNA damage processing and tolerance, DNA repair and DDR, post-damage cell fate determination, intratumoral heterogeneity, and tumor microenvironmental modulation.

While DNA DSB repair and DDR have traditionally been regarded as a central mechanistic axis of radioresistance, the preceding modules demonstrate that repair capacity operates within broader regulatory contexts involving redox balance, metabolic state, and mitochondrial function. Downstream modules further determine whether damaged cells are eliminated or persist, and whether resistant subpopulations expand under ecological constraints.

This layered classification clarifies that radioresistance is not a single molecular abnormality but a structured, hierarchical process in which damage induction, processing, repair, and survival are integrated across multiple biological scales. By aligning these modules with the conceptual framework established for chemoresistance, this organization provides a foundation for understanding mechanistic intersections and modality-specific determinants of treatment failure.

## 6 Drug-specific architectures of chemoresistance

**Figure 3:**
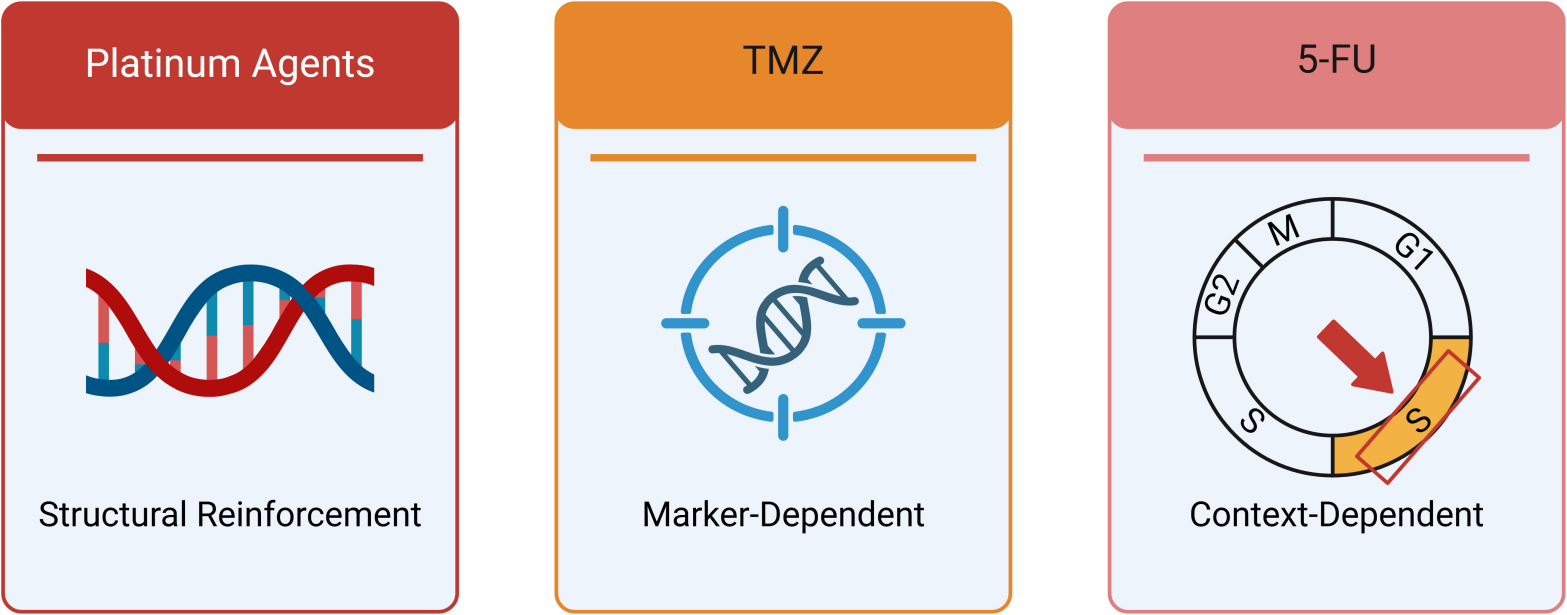
Distinct paradigms governing therapeutic response and resistance across three chemotherapeutic classes. Platinum agents are depicted as “structural reinforcement” drugs whose cytotoxicity and resistance are largely shaped by stable, multi-layered cellular programs, including DNA repair capacity, redox regulation, drug detoxification, transporter-mediated efflux, and stress tolerance. TMZ represents a “marker-dependent” paradigm in which treatment response is strongly modulated by defined predictive biomarkers, most notably MGMT status. In contrast, 5-FU is illustrated as “context-dependent” because its efficacy and resistance are highly influenced by proliferative state, cell-cycle context, nucleotide metabolism, and tumor-specific signaling states. This framework emphasizes that different chemotherapeutic classes impose distinct selective pressures and generate distinct resistance architectures, which may nevertheless partially converge with radioresistance through shared adaptive biological programs.

To orient the discussion in this section, we summarize three representative drug-class–specific resistance paradigms—platinum agents (structural reinforcement), TMZ (marker-dependent resistance), and 5-FU (context-dependent adaptation)—as illustrated in **Figure 3**. These paradigms highlight distinct initiating mechanisms and adaptive trajectories underlying chemoresistance across major CRT backbone agents.

### 6.1 Platinum-based agents

#### 6.1.1 Overview

Platinum-based agents constitute a major class of DNA-damaging cytotoxic drugs widely used in the treatment of solid malignancies. These compounds share a central platinum core that, following intracellular activation, forms covalent DNA adducts, thereby disrupting replication and transcription and ultimately inducing cell death (84, 85). Among them, cisplatin represents the prototypical platinum agent and serves as a backbone of concurrent CRT across multiple tumor types, including cervical, head and neck, lung, and esophageal cancers (86, 87). Clinically, cisplatin is frequently administered in locally advanced disease settings, where it contributes to improved locoregional control and survival outcomes. Nevertheless, therapeutic benefit remains limited in a substantial subset of patients due to primary non-response or the development of acquired resistance after repeated exposure, leading to relapse and poor prognosis (86–88).

Resistance to cisplatin arises through multilayered mechanisms involving altered drug transport and detoxification, enhanced DNA repair and damage tolerance, and stabilization of therapy-tolerant subpopulations under microenvironmental selection pressure (89). Carboplatin is commonly used as an alternative platinum agent in CRT regimens, whereas oxaliplatin is primarily employed in systemic chemotherapy settings and has shown limited or context-dependent benefit in concurrent CRT strategies. Although all three agents generate cytotoxic DNA adducts, differences in chemical structure may influence adduct recognition, repair pathway engagement, and the resistance architectures that become stabilized under therapeutic pressure (84, 85).

Given their frequent integration with radiotherapy, platinum resistance mechanisms may influence therapeutic responsiveness within CRT settings. The relationship between platinum resistance and response is discussed further in Section 7.

#### 6.1.2 Pharmacological effects

Cisplatin exerts its antitumor activity primarily through the formation of covalent DNA adducts, including intra- and inter-strand crosslinks that disrupt replication and transcription and activate the DDR (90). Among these lesions, 1,2-intrastrand crosslinks between adjacent guanine residues represent the predominant cytotoxic adduct species. During DNA replication, platinum-induced crosslinks stall replication forks and generate replication stress. Processing of these lesions requires coordinated engagement of nucleotide excision repair (NER), HR, and translesion synthesis pathways. When repair capacity is exceeded or damage processing becomes dysregulated, replication fork collapse leads to the accumulation of DNA DSBs, thereby converting platinum-induced lesions into lethal cytotoxic signals (91, 92). Thus, platinum cytotoxicity is strongly influenced by the balance between DNA damage formation, repair capacity, and damage tolerance mechanisms.

Carboplatin and oxaliplatin similarly generate platinum–DNA adduct that activate DDR networks, although structural differences influence adducts recognition and processing (93). Carboplatin exhibits slower DNA binding kinetics than cisplatin, with delayed formation and accumulation of DNA adducts consistent with its lower intrinsic reactivity (94, 95). Oxaliplatin, in addition to forming DNA crosslinks, has been reported to disrupt nucleolar organization and suppress RNA polymerase I–mediated rRNA transcription through modification of nucleolar proteins such as fibrillarin (96).

Collectively, the pharmacologic effects of platinum agents converge on replication stress and DSB generation, positioning DDR capacity as a central determinant of therapeutic outcome and as a potential intersection with radiation-induced cytotoxic pathways. In addition to canonical nuclear DNA crosslinking, cisplatin has also been associated with alterations in cellular redox balance and mitochondrial function. Although the relative contribution of these effects to cytotoxicity remains under investigation, oxidative stress responses may represent an additional layer of interaction with radiation-induced ROS. From a conceptual standpoint, such redox-mediated crosstalk has been proposed as a potential contributor to cisplatin–radiotherapy synergy beyond direct DNA damage mechanisms (97).

#### 6.1.3 Position in CRT

Cisplatin-based concurrent CRT represents a standard-of-care approach across multiple solid tumors, including locally advanced cervical, head and neck, and esophageal cancers, as well as stage III NSCLC (87, 88). Randomized trials have demonstrated improved locoregional control and survival outcomes with cisplatin-containing CRT compared with radiotherapy alone, establishing cisplatin as a central backbone of combined-modality therapy in these disease contexts. The molecular rationale for this strategy lies in the complementary induction of DNA damage. Ionizing radiation induces immediate DNA DSBs, whereas cisplatin generates DNA crosslinks that stall replication and, upon processing, can give rise to DSBs through replication fork collapse. Although the initiating lesions differ, both modalities ultimately converge on DSB accumulation and activation of DDR pathways. Concurrent administration increases cumulative genotoxic stress and may enhance fixation of irreparable lesions, thereby improving tumor control.

Experimental and clinical observations suggest that radiosensitization is enhanced when cisplatin administration temporally overlaps with radiotherapy, whereas prolonged intervals may attenuate synergistic effects, as supported by preclinical and clinical reports (97). These findings support a time-dependent interaction between platinum-induced stress responses and radiation-induced damage.

In clinical practice, platinum selection is often individualized. Carboplatin is frequently used as an alternative when cisplatin-related toxicities—such as nephrotoxicity or neurotoxicity—limit tolerability, whereas oxaliplatin is more commonly deployed within systemic chemotherapy backbones and has shown limited or context-dependent benefit in concurrent CRT strategies (98).

Despite its established role, a recurrent clinical challenge is that tumors persisting after platinum-based CRT often exhibit multilayered adaptive programs involving altered drug exposure, stress buffering, and survival signaling convergence (99–101).

#### 6.1.4 Resistance mechanisms

Platinum resistance reflects multilayered adaptive programs that extend beyond single-gene alterations. The mechanisms summarized below are supported to varying degrees by *in vitro* studies, animal models, and clinical tumor analyses; therefore, experimentally defined resistance modules are distinguished from clinically validated predictors of treatment response where applicable. Although cisplatin, carboplatin, and oxaliplatin differ in lesion chemistry and pharmacokinetics, resistance frequently converges on shared regulatory modules that govern intracellular exposure, detoxification, lesion recognition, DNA damage processing, cell fate control, clonal evolution, and microenvironmental reinforcement.

##### 6.1.4.1 Module 1: effective drug delivery

A primary resistance layer involves reduced effective intracellular platinum accumulation. Downregulation of uptake transporters such as CTR1 limits cisplatin entry (102), whereas upregulation of efflux systems—including ABC transporters (e.g., MRP family) (103) and copper-transporting ATPases (ATP7A/ATP7B)—enhances drug extrusion. In ovarian carcinoma models, ATP7B overexpression decreases intracellular carboplatin levels by restricting early accumulation and accelerating export (104). For oxaliplatin, MRP2/ABCC2 expression correlates with non-response, and experimental overexpression reduces intracellular platinum content and cytotoxicity, whereas knockdown restores sensitivity (105). Exposure control may become transcriptionally stabilized through upstream stress regulators such as YB-1 and APE1 (106–108), linking drug transport modulation to broader adaptive programs.

##### 6.1.4.2 Module 2: damage chemistry and redox control

Beyond transport, platinum tolerance is reinforced by glutathione-centered detoxification systems. Elevated GST-π/GSH-associated conjugation neutralizes electrophilic platinum intermediates and reduces effective DNA binding (109). Resistance frequently involves activation of NRF2-driven antioxidant networks, including HO-1, NQO1, and GCLC (110). These pathways constitute integrated redox-buffering architectures that attenuate oxidative stress responses induced by platinum exposure. Enhanced redox buffering may also modulate cellular tolerance to radiation-generated ROS, thereby reinforcing stress tolerance under genotoxic conditions.

##### 6.1.4.3 Module 3: lesion formation and damage processing

Platinum cytotoxicity depends on the formation, recognition, and persistence of platinum–DNA adducts (84, 111). Alterations affecting adduct formation kinetics, chromatin accessibility, or lesion recognition by DNA damage sensors—including high-mobility group (HMG) domain proteins and NER recognition factors—can modulate drug–target interaction efficiency (84, 112). Differences in adduct structure—particularly those associated with oxaliplatin’s DACH ligand—may influence sensor binding affinity and downstream signaling, shifting the balance between effective lesion interpretation and subsequent repair engagement (93). Reduced adduct persistence or altered recognition may therefore diminish cytotoxic signaling even when intracellular drug levels are adequate (84, 111).

##### 6.1.4.4 Module 4: DNA damage processing and repair

Reinforced DNA damage processing represents a major resistance module (85). Upregulation of NER and HR together with enhanced translesion synthesis enables more efficient removal or bypass of platinum-induced crosslinks. Sustained checkpoint activation and replication fork stabilization reduce conversion of stalled forks into lethal DNA DSBs, a process mechanistically linked to replication stress responses (113). Reinforcement of DDR capacity attenuates platinum cytotoxicity by limiting lethal DSB accumulation.

##### 6.1.4.5 Module 5: cell fate determination

Even when DNA damage accumulates, resistance may arise from altered cell fate signaling (85). Dysregulation of p53-dependent apoptosis, enhanced pro-survival signaling (e.g., PI3K/AKT), and checkpoint adaptation can permit survival despite persistent genotoxic stress (114). Such rewiring shifts the balance from apoptosis toward transient arrest, senescence, or reversible quiescence, allowing tumor cells to tolerate platinum-induced damage and subsequently re-enter proliferative states.

##### 6.1.4.6 Module 6: clonal selection and plasticity

Cisplatin resistance is frequently associated with enrichment of stem-like or therapy-tolerant subpopulations (115). Coupling between epithelial-mesenchymal transition (EMT) and stemness stabilizes survival programs under genotoxic pressure (116). These subclones often display enhanced DNA repair capacity, metabolic plasticity, and antioxidant buffering. Clonal selection under platinum pressure thereby reshapes tumor architecture toward more resilient cellular states.

##### 6.1.4.7 Module 7: microenvironmental integration

The tumor microenvironment further stabilizes resistance phenotypes (117). Hypoxia, stromal signaling, inflammatory cytokines, and extracellular matrix remodeling can reinforce survival pathways and modulate drug distribution. Hypoxia-driven signaling may enhance DNA repair proficiency and antioxidant capacity (118), while stromal-derived growth factors sustain pro-survival signaling cascades. These external cues integrate with intrinsic resistance modules, amplifying tolerance under concurrent CRT.

### 6.2 TMZ

#### 6.2.1 Overview

TMZ is a second-generation imidazotetrazine alkylating agent and constitutes the backbone of standard therapy for newly diagnosed adult glioblastoma (119, 120). It is an orally administered small-molecule compound that undergoes spontaneous hydrolysis at physiological pH to generate the active metabolite 5-(3-methyltriazen-1-yl) imidazole-4-carboxamide (MTIC) (121). This chemical activation does not require hepatic enzymatic metabolism. In addition, TMZ readily penetrates the blood–brain barrier (122), enabling therapeutically relevant concentrations within the central nervous system.

Clinically, TMZ is administered concomitantly with radiotherapy followed by adjuvant maintenance therapy as part of the so-called Stupp regimen (10). This combined CRT strategy remains the only treatment approach that has demonstrated a significant survival benefit in patients with glioblastoma and continues to represent the international standard of care.

TMZ exerts its antitumor effect through DNA methylation. It predominantly methylates guanine at the N7 and O^6^ positions and adenine at the N3 position (120). Among these lesions, O^6^-methylguanine—although generated at relatively low frequency—represents the critical cytotoxic lesion, as it drives replication-dependent DNA damage and cell death. The antitumor efficacy of TMZ is therefore strongly influenced by tumor DNA repair capacity, particularly the expression of MGMT. Promoter methylation of the MGMT gene is associated with reduced MGMT expression and correlates with improved treatment response and favorable prognosis (40).

#### 6.2.2 Pharmacological effects

The antitumor activity of TMZ depends on the conversion of DNA methylation damage into irreversible cytotoxic signals. TMZ induces multiple DNA adducts, predominantly N7-methylguanine, N3-methyladenine, and O^6^-methylguanine. Among these, O^6^-methylguanine—although formed at lower frequency—is the principal cytotoxic lesion (123). During DNA replication, O^6^-methylguanine mispairs with thymine. This mismatch is recognized by the mismatch repair (MMR) system. However, because the lesion resides on the template strand, it cannot be removed by MMR. Therefore, repeated attempts at repair generate a so-called futile repair cycle, leading to the accumulation of single-strand breaks (SSBs), replication fork collapse, and ultimately DNA DSBs (124–126). Thus, TMZ cytotoxicity is both replication-dependent and MMR-dependent. Consistent with this mechanism, loss of MMR function confers resistance to TMZ (127).

In addition to its canonical DNA damage mechanism, TMZ has also been reported to influence mitochondrial function and cellular redox status. Alterations in mtDNA maintenance, respiratory activity, and ROS production have been observed following TMZ exposure in glioma models and may contribute to stress signaling and cell death responses (128–130). Mitochondrial-associated apoptotic pathways and mitophagy-related mechanisms have likewise been implicated in modulation of TMZ sensitivity (131, 132).

#### 6.2.3 Position in CRT

TMZ demonstrated a clear survival benefit in glioblastoma when administered as part of concurrent CRT with radiotherapy. The regimen consisting of radiotherapy with concomitant TMZ followed by adjuvant maintenance TMZ—the so-called Stupp regimen—remains the only treatment strategy that has shown a significant overall survival advantage in a phase III randomized trial and continues to represent the international standard of care for newly diagnosed glioblastoma (10, 133). The molecular rationale for this approach lies in the combined induction of replication-associated and radiation-induced DNA damage. Radiotherapy induces immediate DNA DSBs through ionizing radiation, whereas TMZ generates O^6^-methylguanine lesions that, during DNA replication, are converted into DSBs in a replication- and MMR-dependent manner (134). Although the initial lesions differ, both treatments ultimately promote DSBs accumulation and excessive genotoxic stress, leading to cell death. Concurrent administration increases the overall DNA damage burden, thereby promoting fixation of irreparable cytotoxic lesions (135).

In addition, TMZ is associated with relatively manageable systemic toxicity and can be administered daily during radiotherapy, facilitating treatment continuity. Its therapeutic efficacy can also be stratified according to MGMT promoter methylation status, which predicts responsiveness to treatment. For these reasons, TMZ is regarded as a central therapeutic component of CRT for glioblastoma.

#### 6.2.4 Resistance mechanisms

Resistance to TMZ arises through multilayered mechanisms involving enhanced DNA repair, tolerance of DNA damage, altered damage response signaling, and metabolic adaptation. These processes limit the efficacy of both TMZ monotherapy and TMZ-based CRT.

##### 6.2.4.1 Module 1: Effective damage delivery

TMZ readily penetrates the blood–brain barrier; therefore, primary resistance at this level is less commonly attributable to impaired drug delivery. However, altered tumor perfusion or microenvironmental factors may influence effective drug concentration in specific contexts.

##### 6.2.4.2 Module 2: damage chemistry and redox control

TMZ-resistant cells frequently display altered mitochondrial bioenergetics, increased oxidative phosphorylation capacity, and upregulation of antioxidant systems such as SOD2 and glutathione metabolism (128). Enhanced detoxification of ROS mitigates oxidative stress induced by TMZ and radiotherapy, thereby reinforcing survival (136, 137).

##### 6.2.4.3 DNA damage recognition and processing

Acquired resistance frequently involves defects in the MMR pathway (e.g., loss of MSH6 or MLH1), preventing replication-dependent processing of O^6^-methylguanine into DSBs (127). As a result, DNA methylation damage becomes less efficiently linked to downstream cytotoxic signaling.

##### 6.2.4.4 Module 4: DNA damage processing and repair

The principal mechanism of primary resistance is overexpression of MGMT, which directly removes O^6^-methylguanine lesions before they are converted into lethal DNA damage (40, 138). Upregulation of base excision repair (BER), particularly Poly (ADP-ribose) polymerase (PARP) -dependent SSB repair, further enhances tolerance to TMZ-induced N7- and N3-methyl lesions (139).

##### 6.2.4.5 Module 5: cell fate determination

TMZ-resistant glioma cells exhibit sustained activation of ATR–CHK1 signaling and G2/M checkpoint pathways, which prolong DNA repair time and protect cells from mitotic catastrophe. Dysregulation of p53 and checkpoint control further enhances survival under genotoxic stress (140–142).

##### 6.2.4.6 Module 6: clonal selection and plasticity

Glioma stem-like cells (GSCs), characterized by strong DNA repair capacity, metabolic flexibility, and robust antioxidant defenses, are preferentially selected during treatment and contribute to tumor recurrence (44).

##### 6.2.4.7 Module 7: microenvironmental integration

Hypoxic niches and microenvironmental constraints may further modulate TMZ responsiveness by altering proliferation rates, redox balance, and DNA repair capacity. Collectively, TMZ resistance reflects coordinated reprogramming of DNA repair networks, cell-cycle regulation, mitochondrial metabolism, and redox homeostasis.

### 6.3 5-FU

#### 6.3.1 Overview

5-FU is a fluorinated pyrimidine antimetabolite that exerts cytotoxic activity through disruption of nucleotide metabolism and nucleic acid synthesis. Its active metabolite FdUMP (fluorodeoxyuridine monophosphate) forms a stable ternary complex with thymidylate synthase and reduced folates, thereby inhibiting dTMP synthesis and impairing DNA replication. In parallel, FUTP (fluorouridine triphosphate) can be incorporated into RNA, disrupting RNA processing and translation, while FdUTP misincorporation into DNA contributes to DNA damage and replication stress (143). Through these combined effects on DNA and RNA metabolism, 5-FU predominantly affects rapidly proliferating tumor cells.

Clinically, 5-FU has long served as a backbone drug in the treatment of solid tumors, particularly gastrointestinal malignancies such as colorectal, gastric, pancreatic, and esophageal cancers, where it is frequently incorporated into multi-agent regimens and concurrent CRT protocols. Owing to its capacity to perturb nucleotide pools and enhance replication-associated DNA damage, 5-FU has historically been regarded as a radiosensitizing agent in combined-modality therapy.

Pharmacokinetically, 5-FU is typically administered intravenously because of rapid hepatic degradation by dihydropyrimidine dehydrogenase (DPD), resulting in a short plasma half-life but sustained antitumor effects under continuous infusion schedules (144). Interindividual variability in DPD activity influences both systemic exposure and toxicity, and may contribute to heterogeneity in therapeutic response. Despite its broad clinical applicability, clinical benefit is often limited by primary non-response and acquired resistance, driving ongoing optimization of dosing strategies and combination approaches.

#### 6.3.2 Pharmacological effects

5-FU exerts antitumor activity through intracellular conversion to FdUMP, FUTP, and FdUTP, thereby targeting both DNA and RNA metabolism. A central DNA-directed mechanism is thymidylate synthase inhibition by FdUMP, which depletes dTMP/dTTP pools and induces “thymineless stress.” This nucleotide imbalance leads to stalled replication forks, misincorporation events, and activation of DDR signaling pathways, including ATR/CHK1, which can culminate in apoptosis or mitotic catastrophe (145, 146). This DNA-directed pathway is widely regarded as the dominant and best-validated cytotoxic mechanism of 5-FU. In parallel, misincorporation of FdUTP into DNA promotes BER cycling, generating persistent SSBs. During S phase, accumulation of unrepaired SSBs can trigger replication fork collapse and secondary DNA DSBs, thereby amplifying replication-associated cytotoxic stress. These effects are strongly cell-cycle dependent, with maximal vulnerability in actively proliferating, S-phase–enriched tumor fractions. Most supporting evidence derives from experimental and preclinical systems, although clinical correlations have been reported. RNA-directed toxicity also contributes to antitumor activity. FUTP incorporation into RNA perturbs rRNA and tRNA processing and disrupts translational fidelity, reinforcing proteotoxic and nucleolar stress responses (147). The relative quantitative contribution of RNA-directed toxicity to overall clinical efficacy remains incompletely defined.

Overall therapeutic efficacy reflects the balance between metabolic activation versus inactivation (notably DPD-mediated catabolism), the magnitude of replication stress and damage signaling, and the integrity of stress-execution pathways such as p53-dependent apoptosis (143). Because 5-FU–induced cytotoxicity is closely coupled to replication-associated DNA damage and checkpoint activation, it can enhance radiosensitivity during concurrent CRT treatment.

#### 6.3.3 Position in CRT

In CRT, 5-FU functions not merely as a cytotoxic agent but as a central radiosensitizing component. Continuous infusion of 5-FU is widely employed in neoadjuvant CRT for rectal cancer, and in esophageal cancer it is frequently combined with platinum-based agents within multimodal treatment regimens (148–150). In these contexts, 5-FU has been shown to contribute substantially to local tumor control and pathological response.

Mechanistically, sustained thymidylate synthase inhibition under continuous infusion maintains S-phase–associated “thymineless” replication stress. This nucleotide imbalance is thought to impair efficient repair of radiation-induced DNA lesions and increases the likelihood that such lesions become irreversibly fixed as lethal damage (151). Through this replication-dependent mechanism, 5-FU amplifies radiation-induced cytotoxicity without necessarily sharing the same primary DNA lesion. This model is supported primarily by preclinical mechanistic studies, with indirect validation from clinical response patterns.

Clinically, the feasibility of daily administration during radiotherapy supports treatment continuity and maintains consistent radiosensitizing pressure (15). Consequently, the therapeutic benefit of 5-FU–based CRT is generally attributed to enhanced replication stress and increased fixation of radiation-associated DNA damage rather than chemotherapy-driven tumor shrinkage alone.

#### 6.3.4 Resistance mechanisms

5-FU resistance develops through coordinated adaptive programs that attenuate replication stress, buffer oxidative damage, and prevent lethal damage fixation. Consistent with the modular framework applied to TMZ and platinum agents, resistance can be conceptualized across seven hierarchical layers.

##### 6.3.4.1 Module 1: effective drug delivery and metabolic gating

A core resistance layer involves altered intracellular handling of 5-FU that reduces net production of toxic metabolites (FdUMP/FUTP/FdUTP). Increased catabolism via DPD/DPYD upregulation, reduced activation through pyrimidine-salvage enzymes, and nucleotide pool rebalancing that buffers thymidylate synthase inhibition can markedly lower functional drug activity even when systemic exposure is adequate (152–155). Through such metabolic gating, cells attenuate replication stress at its source.

##### 6.3.4.2 Module 2: damage chemistry and redox control

Although 5-FU primarily induces replication-associated stress rather than direct oxidative DNA damage, sustained nucleotide imbalance and RNA perturbation may secondarily affect mitochondrial function and cellular redox balance. Experimental studies suggest that prolonged replication stress can be accompanied by increased ROS production, particularly under conditions of metabolic strain. In resistant models, adaptive upregulation of antioxidant pathways—including NRF2-associated transcriptional programs, glutathione metabolism, and superoxide dismutase activity—has been reported (156). These changes may enhance tolerance to oxidative stress and indirectly limit amplification of replication-associated damage. However, compared with platinum agents or radiation, redox remodeling is generally considered a modulatory rather than dominant mechanism of 5-FU resistance. In the context of CRT, such antioxidant adaptation may enhance tolerance to oxidative stress under therapy-induced metabolic strain.

##### 6.3.4.3 Module 3: lesion formation and damage processing

Resistance may also involve altered cellular responses to thymidylate synthase inhibition and nucleic-acid misincorporation. Nucleotide pool rebalancing and increased TS expression can attenuate the biochemical consequences of dTMP depletion, thereby reducing the magnitude of replication stress rather than completely preventing DNA damage (155). In addition, tolerance of uracil or mis-incorporated bases may limit propagation of replication-associated lesions. Rather than fully preventing DNA damage, these adaptations may dampen the signaling intensity linking nucleotide imbalance to checkpoint activation and downstream cytotoxic pathways (157). As a result, tumor cells may partially uncouple metabolic disruption from lethal damage execution.

##### 6.3.4.4 Module 4: DNA damage processing and repair

Enhanced DNA repair and tolerance pathways further stabilize survival under 5-FU–induced stress. Upregulation of BER, increased PARP-dependent SSB repair, and reinforcement of ATR/CHK1-mediated replication fork protection reduce the probability of fork collapse and secondary DSB formation (145, 151). These repair adaptations blunt lethal damage fixation.

##### 6.3.4.5 Module 5: cell fate determination

Checkpoint consolidation and anti-apoptotic bias determine whether replication stress culminates in cell death or survival. Sustained ATR/CHK1 signaling, skewing of BCL-2 family regulators, adaptive autophagy, and context-dependent alterations in p53 responses collectively shift the damage–decision threshold toward tolerance rather than apoptosis or mitotic catastrophe (145, 158). This buffering of cell fate determination reduces effective cytotoxic conversion without necessarily altering upstream lesion formation or repair capacity.

##### 6.3.4.6 Module 6: clonal selection and plasticity

Under treatment pressure, colorectal cancer cells have been reported to adopt persister-like or stem-like states characterized by relative quiescence, metabolic flexibility, and enhanced repair capacity. Enrichment of CSC-like populations such as CD133^+^CD24^lo cells and EMT-associated transcriptional programs (e.g., SNAI2–miR-145 axis) has been implicated in experimental models as contributors to reversible stress tolerance and subsequent regrowth (159–161). Such plasticity suggests that, in at least a subset of contexts, resistance may reflect adaptive transcriptional reprogramming rather than exclusively fixed mutational events.

##### 6.3.4.7 Microenvironmental and epigenetic contributions

Tumor microenvironmental constraints—including hypoxia, nutrient limitation, and stromal signaling—have been reported to influence proliferation rates, redox balance, and DNA repair capacity, thereby modulating 5-FU responsiveness. Concurrently, epigenetic remodeling may contribute to the stabilization of therapy-persistent states through coordinated DNA methylation changes, histone modification programs, and chromatin accessibility shifts (162, 163). Integrated chromatin and transcriptomic profiling in experimental models suggest that resistance-associated networks can become partially stabilized at the chromatin level, potentially supporting durable adaptation even after drug withdrawal.

## 7 Convergence between chemoresistance and radioresistance

Although platinum agents, TMZ, and 5-FU induce distinct primary forms of cellular injury, resistant tumors frequently exhibit partial convergence on shared adaptive mechanisms involving reinforcement of DNA damage processing, checkpoint stabilization, redox buffering, metabolic adaptation, and therapy-tolerant plasticity. These overlapping adaptive programs may, under certain conditions, reduce responsiveness to both chemotherapy and radiotherapy, thereby contributing to treatment persistence and recurrence following CRT.

Importantly, convergence does not imply uniform or complete cross-resistance. Rather, current evidence suggests that resistance propagation is context-dependent and frequently restricted to specific adaptive axes shaped by lesion type, repair pathway utilization, metabolic state, and tumor lineage. Accordingly, cross-resistance should not be interpreted as a simple consequence of shared DNA damage, but instead as the emergent outcome of partially overlapping adaptive architectures.

In the following sections, we discuss the shared mechanisms potentially linking chemoresistance and radioresistance, the heterogeneous nature of cross-resistance phenotypes, and the translational implications of targeting convergent adaptive vulnerabilities in CRT-resistant tumors.

### 7.1 Shared adaptive convergence

**Figure 4:**
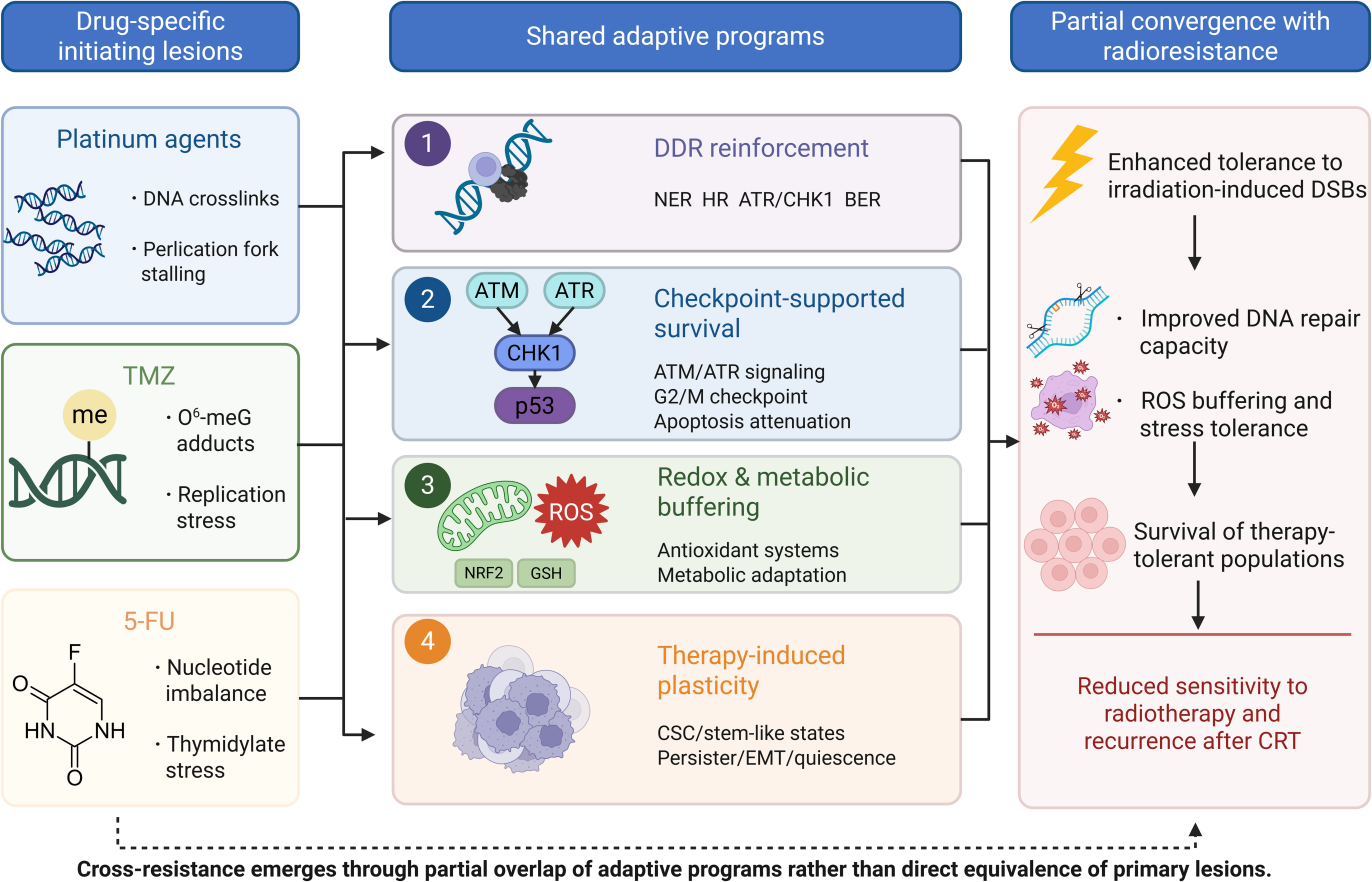
Shared adaptive convergence linking chemoresistance and radioresistance. This schematic illustrates how distinct chemotherapeutic classes may initiate resistance through different primary lesions but converge on shared adaptive programs that partially overlap with radioresistance. Platinum agents, TMZ, and 5-FU are shown as representative drug classes that induce different forms of cellular stress, including DNA crosslinks, O^6^-methylguanine adducts, replication stress, nucleotide imbalance, and thymidylate stress. Despite these drug-specific initiating mechanisms, resistant cells may acquire common adaptive features, including DDR reinforcement, checkpoint-supported survival, redox and metabolic buffering, and therapy-induced plasticity. These shared programs can enhance DSB tolerance, improve DNA repair capacity, increase ROS buffering and stress tolerance, and promote the survival of therapy-tolerant populations. The model emphasizes that cross-resistance between chemotherapy and radiotherapy arises from partial overlap of adaptive mechanisms rather than direct equivalence of primary DNA lesions.

Shared convergence between chemoresistance and radioresistance is most frequently observed across four adaptive layers: DDR reinforcement, checkpoint-supported survival, redox/metabolic buffering, and therapy-induced plasticity (**Figure 4**).

#### 7.1.1 Reinforcement of DNA damage processing and repair capacity (Module 4)

Although platinum agents, TMZ, and 5-FU induce distinct primary lesions, resistance to these therapies frequently converges on enhanced DDR capacity. In platinum-resistant tumors, enhanced NER and HR attenuate DNA crosslink persistence and reduce lethal DSB accumulation (84, 85). TMZ resistance similarly involves MGMT-dependent lesion reversal, altered MMR-associated processing, and increased HR activity (40, 127, 164). In 5-FU–resistant models, activation of ATR/CHK1-mediated replication surveillance and HR pathways reduces replication fork collapse and secondary DSB formation (145, 151). Collectively, these observations suggest that reinforcement of DDR capacity may represent a partially shared adaptive mechanism linking chemoresistance and radioresistance.

#### 7.1.2 Sustained checkpoint signaling and cell fate adaptation (Modules 4–5)

Sustained checkpoint signaling and altered cell fate determination represent a second layer of convergence between chemoresistance and radioresistance. Across platinum-, TMZ-, and 5-FU–resistant models, persistent activation of ATM/ATR-associated DDR pathways has been linked to prolonged damage tolerance and attenuation of apoptosis following genotoxic injury. Platinum resistance is associated with ATM/ATR activation and attenuation of p53-mediated apoptosis (84, 114, 165), TMZ resistance with constitutive ATR–CHK1 signaling and G2/M checkpoint persistence (140–142), and 5-FU resistance with reinforcement of ATR/CHK1-mediated replication surveillance and S-phase checkpoint stabilization (145–147). Because these checkpoint pathways are also central regulators of radiation-induced DDR, persistent checkpoint-supported survival may constitute a partially shared adaptive mechanism linking chemoresistance and radioresistance (165–167).

#### 7.1.3 Redox and metabolic remodeling (Module 2)

Redox buffering and metabolic adaptation represent an additional layer of convergence between chemoresistance and radioresistance. Resistant tumors frequently exhibit adaptive remodeling of antioxidant systems and metabolic stress responses that may enhance tolerance to therapy-induced oxidative stress. Platinum resistance is associated with NRF2-driven antioxidant programs and glutathione-dependent detoxification pathways (84, 109, 110), whereas TMZ-resistant glioma models demonstrate increased antioxidant capacity, including SOD2 upregulation and catalase-associated detoxification systems (128, 168, 169). Compared with platinum agents and TMZ, redox remodeling appears to play a less dominant role in 5-FU resistance, although prolonged exposure has been associated with OXPHOS-dependent and antioxidant-buffered cellular states in experimental models (170, 171). Collectively, these findings suggest that adaptive redox remodeling may represent a partially shared mechanism linking chemoresistance and radioresistance.

#### 7.1.4 Clonal selection and plasticity (Module 6)

Clonal selection and therapy-induced plasticity represent an additional convergence layer linking chemoresistance and radioresistance. Across platinum-, TMZ-, and 5-FU–resistant models, treatment pressure has been associated with enrichment of tumor subpopulations characterized by enhanced DNA repair capacity, metabolic flexibility, stress tolerance, and relative quiescence. Platinum resistance may select for CSC-like subpopulations with enhanced DDR activity and antioxidant buffering (85, 89, 115, 116), TMZ resistance for glioma stem-like cells with strong repair and checkpoint capacity (44), and 5-FU resistance for persister-like or EMT-associated states linked to reversible therapy tolerance (159–161). Collectively, these findings suggest that therapy-driven plasticity may represent a partially shared adaptive mechanism linking chemoresistance and radioresistance.

### 7.2 Heterogeneous and axis-restricted cross-resistance

Across experimental models, cross-resistance between chemotherapy and radiotherapy does not follow a uniform or hierarchical pattern. Instead, reciprocal but incomplete relationships are frequently observed, indicating that resistance propagation is axis-restricted rather than modality-determined.

In some systems, radioresistance co-emerges with cisplatin resistance, particularly in models established through repeated high-dose irradiation (172). Such phenotypes are consistent with reinforcement of DNA damage processing and checkpoint governance modules, which overlap functionally with platinum-induced replication stress resolution. However, other radioresistant models preferentially exhibit 5-FU resistance without altered cisplatin sensitivity (173), suggesting convergence along replication-stress adaptation and metabolic gating axes rather than crosslink-specific processing pathways.

Conversely, resistance initiated by chemotherapeutic pressure does not uniformly confer radioresistance. Etoposide-resistant cells, for example, may display enhanced tolerance to radiation while retaining unchanged sensitivity to cisplatin and 5-FU (174), reflecting a DSB-processing–restricted adaptation. In contrast, certain cisplatin-resistant states exhibit concomitant resistance to 5-FU yet fail to acquire radioresistance (175), consistent with platinum-handling–dominant mechanisms that do not necessarily enhance radiation-relevant damage processing. In a murine L5178Y model, 5-FU–resistant cells were cross-resistant to cisplatin but not to ionizing radiation, further illustrating that replication-stress adaptation may propagate toward platinum tolerance without conferring enhanced DSB repair competence (176). Similarly, TMZ-resistant glioma models do not uniformly demonstrate increased radioresistance (177), underscoring that marker-dependent alkylation repair mechanisms (e.g., MGMT-mediated repair) do not automatically translate into strengthened DSB repair competence.

Additional complexity arises from alterations in radiation survival curve architecture. Some cisplatin-resistant lines exhibit expansion of the low-dose shoulder without substantial changes in terminal slope, indicating enhanced sublethal damage handling rather than global shifts in intrinsic radiosensitivity (178). Moreover, enrichment of stem-like or therapy-tolerant subpopulations has been associated with resistance to both 5-FU and radiotherapy in several tumor contexts (179, 180), yet such cell-state enrichment is not universally observed across all resistant models. For example, glioblastoma-derived sphere models enriched for stem-like properties—such as U-251 compared with U-87—have been reported to display relative resistance to both TMZ and radiation, supporting the concept that adaptive cell-state plasticity can simultaneously modulate chemotherapeutic and radiotherapeutic responsiveness (181).

Collectively, these heterogeneous and reciprocal patterns argue against a simple lesion-overlap explanation for cross-resistance. Instead, they suggest that cross-resistance emerges only when selective pressures reinforce partially overlapping resistance mechanisms. Accordingly, cross-resistance appears to depend on the convergence of specific adaptive pathways rather than solely on shared lesion chemistry. These observations support a more integrated mechanistic view of cross-resistance.

### 7.3 Clinical implications and emerging therapeutic strategies

These convergent resistance programs may provide clinically exploitable vulnerabilities. Emerging evidence suggests that adaptive mechanisms associated with chemoresistance and radioresistance can generate candidate biomarkers and potential therapeutic dependencies. Current research efforts therefore focus on patient stratification, longitudinal monitoring, and context-dependent therapeutic intervention.

#### 7.3.1 Potential biomarkers

Multiple resistance-associated adaptive programs are currently being explored as potential biomarker frameworks for therapeutic response, recurrence risk, and treatment adaptation. Among these, alterations involving DDR pathways represent some of the most clinically advanced candidates. Mutational profiles involving TP53, KEAP1/NRF2 signaling, and related stress-response pathways have been associated with radioresistance and poor clinical outcome in several malignancies, including lung squamous cell carcinoma (182). Metabolic adaptation has also emerged as a potentially relevant biomarker axis. HIF-1α–dependent glycolytic reprogramming has been implicated in platinum resistance, 5-fluorouracil resistance, and radioresistance across multiple tumor contexts (183, 184). Altered lipid metabolism, including ACLY expression and plasma sphingolipid signatures, has additionally been associated with radiotherapy response and post-treatment recurrence in exploratory clinical studies (185, 186). Liquid biopsy–based approaches, including circulating tumor DNA (ctDNA), extracellular vesicles (EVs), circulating transcripts, and microRNA profiling, are also being investigated as minimally invasive tools for monitoring treatment-associated molecular adaptation. Elevated circulating ABCB1 transcripts during platinum-based chemotherapy have been associated with poor prognosis and metastatic phenotypes in ovarian cancer (187). Similarly, EV-associated biomarkers and hypoxia-related exosomal microRNAs have been linked to radioresistance-associated phenotypes and treatment adaptation in several tumor types (188–190). ctDNA- and circulating nucleic acid–based strategies are also being explored for longitudinal monitoring of recurrence and therapeutic response, although prospective validation remains limited (191, 192).

Collectively, these observations suggest that clinically useful resistance biomarkers may ultimately require integrative, multi-dimensional characterization of adaptive tumor states rather than reliance on single molecular alterations.

#### 7.3.2 Therapeutic strategies

Recognition of convergent adaptive resistance mechanisms has stimulated interest in therapies targeting shared survival pathways. Several such strategies are currently being explored in early-phase clinical studies across multiple tumor types. These include DDR-targeting approaches such as PARP inhibition, ATR/CHK1 inhibition, and combination DNA damage–based therapies in tumors with altered checkpoint or HR pathways. Although exploratory and early clinical studies have demonstrated biological rationale and occasional therapeutic activity in platinum-resistant and TMZ-resistant tumor contexts, clinical efficacy remains inconsistent and context dependent (193, 194). Current clinical efforts are also increasingly exploring therapeutic strategies targeting redox/metabolic adaptation and stemness-associated survival programs. Emerging translational and early-phase clinical studies have investigated modulation of mitochondrial metabolism, oxidative stress buffering systems, and therapy-tolerant stem-like cell populations as potential vulnerabilities contributing to persistent treatment resistance and recurrence (195–198). Additional exploratory approaches have targeted adaptive survival signaling through multiple pathways. Early-phase clinical studies have evaluated PI3K/AKT/mTOR modulation in combination with cisplatin-based CRT to enhance treatment response in head and neck cancer (199), whereas preclinical *in vivo* studies have examined NF-κB–associated stress signaling in cisplatin-resistant tumors (200). Combined *in vitro* and clinical pharmacokinetic studies have also investigated anti-apoptotic BCL-2 pathway targeting to enhance radiation response in head and neck cancer (201). In addition, phase II clinical studies have explored metabolic modulation using metformin as a strategy to suppress CSC–associated phenotypes and potentially enhance platinum sensitivity in ovarian cancer, including increased ex vivo cisplatin sensitivity and reduced CSC-associated populations (202).

Collectively, these studies suggest that adaptive resistance states may provide mechanistically relevant therapeutic opportunities, although most proposed strategies remain exploratory and have not yet demonstrated broadly effective clinical benefit.

#### 7.3.3 Clinical caveat

Despite increasing mechanistic evidence supporting partial convergence between chemoresistance and radioresistance, current clinical assessment of treatment resistance still relies largely on post-treatment tumor response and recurrence monitoring. Prospective clinical studies incorporating longitudinal biomarker acquisition during therapy remain limited. Further progress will likely require integrated clinical datasets capable of capturing adaptive resistance dynamics across tumor types and treatment modalities, including chemotherapy, radiotherapy, and combined CRT settings. Accordingly, most proposed convergence mechanisms are currently supported primarily by mechanistic and preclinical studies, whereas direct clinical evidence for cross-resistance across treatment modalities remains limited.

### 7.4 Convergent adaptive architecture

#### 7.4.1 From lesion-specific models to adaptive infrastructure

Traditional models of therapeutic resistance have focused on lesion-specific mechanisms, such as enhanced repair of platinum-induced DNA crosslinks or restoration of MGMT activity in TMZ-treated tumors. While these lesion-centered frameworks remain fundamentally important, accumulating evidence suggests that durable resistance frequently reflects broader adaptive survival that extend beyond the initiating insult. Across platinum-, TMZ-, and 5-FU–resistant states, tumors often exhibit partially overlapping programs involving DDR reinforcement, checkpoint stabilization, metabolic remodeling, redox buffering, and therapy-tolerant cell-state plasticity.

Rather than representing entirely independent phenomena, chemoresistance and radioresistance may therefore reflect convergence on shared adaptive infrastructures that modulate damage sensing, stress buffering, and survival-state maintenance across treatment modalities. Importantly, such convergence does not necessarily imply universal or stable cross-resistance, but instead suggests partial overlap within context-dependent adaptive infrastructures.

#### 7.4.2 Therapeutic implications: targeting adaptive infrastructure rather than individual lesions

If resistance convergence reflect stabilization of shared adaptive infrastructures rather than isolated lesion-specific repair events, durable therapeutic control may require coordinated disruption of these survival programs. Strategies targeting DDR dependency, metabolic plasticity, redox adaptation, and therapy-tolerant stem-like states have shown mechanistic rationale and exploratory therapeutic potential in selected preclinical and early clinical settings (166, 203). Accordingly, further therapeutic development may benefit from shifting emphasis from isolated lesion-specific intervention toward integrated disruption of adaptive survival architecture. Such approaches could, in principle, reduce the emergence of cross-resistant tumor states following chemotherapy, radiotherapy, or combined CRT.

## 8 Conclusion

CRT resistance should not be understood solely through the lens of lesion-specific repair mechanisms. Although platinum agents, TMZ, and 5-FU generate distinct primary molecular insults, resistant tumors often appear to converge on shared adaptive programs that attenuate lethal damage conversion.

Across drug classes, resistant tumors commonly exhibit reinforced DNA damage processing, sustained checkpoint governance, metabolic reprogramming, antioxidant buffering, and enrichment of therapy-tolerant cell states. Together, these adaptations stabilize survival under multimodal genotoxic stress. These adaptations reduce both chemotherapy-induced cytotoxicity and radiation-induced lethality, thereby enabling partial cross-resistance even in the absence of identical initiating lesions.

Current evidence suggests that mitochondrial functional plasticity and redox homeostasis may contribute to resistance-associated cellular adaptations through interactions with DNA repair pathways, checkpoint regulation, and cell-state plasticity. This integrated perspective suggests that resistance may arise not only from isolated lesion-specific repair events, but also from broader adaptive survival programs involving DDRs, oxidative stress adaptation, and therapy-tolerant cell states.

Emerging clinical and mechanistic studies further suggest that these adaptive programs may provide candidate therapeutic vulnerabilities. Combination approaches incorporating DDR-targeting agents, PI3K/AKT/mTOR pathway inhibition, redox-modulating strategies, metabolic intervention, or stemness-directed therapies have shown potential to enhance chemoradiosensitivity in selected experimental and early clinical settings. In parallel, liquid biopsy–based monitoring approaches, including ctDNA, extracellular vesicle profiling, and circulating resistance-associated transcripts, may support longitudinal assessment of adaptive resistance evolution during treatment.

Collectively, these findings support a conceptual framework in which chemoresistance and radioresistance are linked through partially shared survival architectures rather than entirely independent lesion-specific processes. Future therapeutic development may therefore benefit from integrated strategies that combine context-aware molecular profiling, longitudinal resistance monitoring, and coordinated disruption of adaptive survival programs.
